# Kidney xenotransplantation: Future clinical reality or science fiction?

**DOI:** 10.1111/nhs.12994

**Published:** 2022-11-16

**Authors:** Daniel Rodger, David K. C. Cooper

**Affiliations:** 1 Institute of Health and Social Care, School of Allied and Community Health, London South Bank University, London, UK; 2 Department of Psychological Sciences, Birkbeck, University of London, London, UK; 3 Center for Transplantation Sciences, Department of Surgery, Massachusetts General Hospital/Harvard Medical School, Boston, Massachusetts, USA

**Keywords:** acute kidney failure, kidney diseases, tissue and organ procurement, transplantation, transplants, xenotransplantation

## Abstract

There is a global shortage of organs for transplantation and despite many governments making significant changes to their organ donation systems, there are not enough kidneys available to meet the demand. This has led scientists and clinicians to explore alternative means of meeting this organ shortfall. One of the alternatives to human organ transplantation is xenotransplantation, which is the transplantation of organs, tissues, or cells between different species. The resurgence of interest in xenotransplantation and recent scientific breakthroughs suggest that genetically engineered pigs may soon present a realistic alternative as sources of kidneys for clinical transplantation. It is therefore important for healthcare professionals to understand what is involved in xenotransplantation and its future implications for their clinical practices. First, we explore the insufficiency of different organ donation systems to meet the kidney shortage. Second, we provide a background and a summary of the progress made so far in xenotransplantation research. Third, we discuss some of the scientific, technological, ethical, and public health issues associated with xenotransplantation. Finally, we summarize the literature on the attitudes of healthcare professionals toward xenotransplantation.

## 1 | INTRODUCTION

The lack of suitable donor organs has presented a problem since kidney transplantation first became a reality in 1954, when Dr Joseph Murray performed a kidney transplant between identical twins in Boston in the United States (Merrill et al., 1956). The limited availability of organs has led to consideration of organs from animals as a means of bridging this shortfall.

Over 850 million people globally have some kind of kidney disease and by 2040 it is projected to rise from being the 16th leading cause of years of life lost to the fifth leading cause (Foreman et al., 2018; Jager et al., 2019). Despite recent attempts by many governments to address the organ shortage by making significant and sometimes controversial changes to a country’s organ donation system, there remains an insufficient supply of organs to meet the demand. The consequences of this shortfall are that each year thousands of people die while waiting for a donor organ, or they become too sick to remain on the transplant waiting list. The kidney remains the most transplanted solid organ in both the United Kingdom and the United States, and subsequently those patients who require a kidney transplant would likely benefit from alternatives if they were similarly efficacious.

One alternative in which there has been a resurgence of scientific and medical interest and activity recently is xenotransplantation—the cross-species transplantation of organs, tissues, or cells between different species. First, we explore the inability of current organ donation systems to meet the kidney shortage. Second, we provide background and a summary of the progress made so far in xenotransplantation research. Third, we discuss some of the scientific, technological, ethical, and economic issues associated with xenotransplantation to help assess whether it will one day become part of routine clinical practice. Finally, we provide a summary of the research that explores the attitudes of healthcare professionals toward xenotransplantation.

A major point that should be stressed is that, for the first time in more than 70 years of experience with organ transplantation, xenotransplantation offers us the ability to modify the organ-source *donor* and not just treat the recipient of a graft. This has immense potential for the future of transplantation.

## 2 | ORGAN DONATION SYSTEMS AND THE KIDNEY SHORTAGE

As of July 29, 2022 there were 4 847 patients in the United Kingdom waiting for a kidney transplant (NHS Blood and Transplant, 2021); approximately 250 die each year while waiting for a donor kidney (Kidney Research UK, 2020). Approximately 3 000 kidney transplants take place every year in the United Kingdom and almost 30 000 people are on dialysis (Kidney Care UK, 2022). Currently, the average time a patient is on the transplant waiting list for a deceased donor organ in the United Kingdom is 2.5–3 years. If a living donor can be identified, the transplant can usually be carried out within 3–6 months and is associated with better short- and long-term health outcomes (Winterbottom et al., 2021).

To bridge the shortfall between the demand for kidneys and the available organs, many governments have made significant changes to their organ donation systems. Consequently, several countries have recently introduced new legislation to increase the total number of organs that become available for transplantation and, it is hoped, save hundreds of lives (Bea, 2021; Madden et al., 2020). These legislative changes most commonly include the adoption of an opt-out system rather than an opt-in system of organ donation.

An opt-in organ donation system describes where an individual must “opt in” by joining the organ donor register (ODR) if they wish to donate their organs after death. An opt-out organ donation system differs from this by presuming consent and so there is no requirement to register with the ODR. Someone who does not wish to be an organ donor must therefore register his/her decision to opt out. Opt-out systems are commonly described as either “soft” or “hard.” In a “soft” opt-out system, family members are consulted prior to organ donation and their wishes are honored; in contrast, in a “hard” opt-out system an individual is presumed to have consented to becoming an organ donor unless they previously opted out, and the wishes of the family are not considered.

Despite improvements, it is well established that changing legislation is not a panacea but is only one component in addressing the organ shortage (Willis & Quigley, 2014). Alternative means of obtaining organs should also be sought. Nevertheless, even with significant investment in infrastructure, such as an increased number of intensive care beds, and education, it is likely that people will continue to die each year waiting for a kidney or other organ transplant. Long-term dialysis is an option for patients in renal failure, but a transplant offers superior long-term outcomes in several key areas and remains the gold standard treatment. For example, following kidney transplantation, patients have (i) improved rates of long-term survival; (ii) a reduced risk of stroke, heart failure, and ischemic heart disease; and (iii) clinically meaningful improvements to quality of life (i.e., physical functioning, mental health, bodily pain, and general health) (Tonelli et al., 2011).

It is the prospect of xenotransplantation as a means of alleviating this organ shortfall—whether as a bridge or destination therapy—that has been the primary motivation behind the pioneering research and developments over the last 30 years.

## 3 | HISTORY OF KIDNEY XENOTRANSPLANTATION

The notion of combining animal and human body parts is not new; it has ancient roots and can be found in Hindu, Egyptian, Mesopotamian, Babylonian, Greek, African, Norse, and Roman mythology and folklore in the form of human-animal hybrids, or humans with animal parts, such as the minotaur, mermaid, faun, gorgon, and lamassu (Ahlqvist & Vandkilde, 2018; Syrrou et al., 2021).

Clinical experiments in xenotransplantation have historically been driven by clinical necessity, for instance, in terminally ill patients in a final attempt to prolong life. In 1906, xenotransplantation—the process of transplanting organs or tissue from a non-human species into a human—was attempted by the French surgeon Mathieu Jaboulay (Table 1). Jaboulay conducted the first clinical kidney xenotransplants on two patients suffering from chronic renal failure. The first patient received a pig kidney and the second a goat kidney. Both xenografts initially produced urine but were removed on the third day posttransplant for what today we would consider to be rejection (Rodger & Hurst, 2022). Between 1963 and 1964 surgeon Keith Reemtsma transplanted chimpanzee kidneys into six patients (Reemtsma et al., 1964). Most patients died within several weeks, but one led an active life for 9 months, even returning to work as a schoolteacher before dying relatively suddenly from what was believed to be an acute electrolyte disturbance.

Since the 1980s there has been a move away from the use of non-human primates toward the use of pigs as source animals for clinical xenotransplantation. This was for a variety of reasons that included difficulties in breeding enough source animals, organ size disparities, ethical concerns, and the increased risk of zoonosis. Pigs are now considered the most realistic and viable animal species for use in xenotransplantation for many reasons (Table 2). Moreover, if rejection of a pig organ can be successfully prevented for a long period of time, xenotransplantation has several advantages over allotransplantation (Table 3).

## 4 | EDITING THE PIG GENOME

**FIGURE 1:**
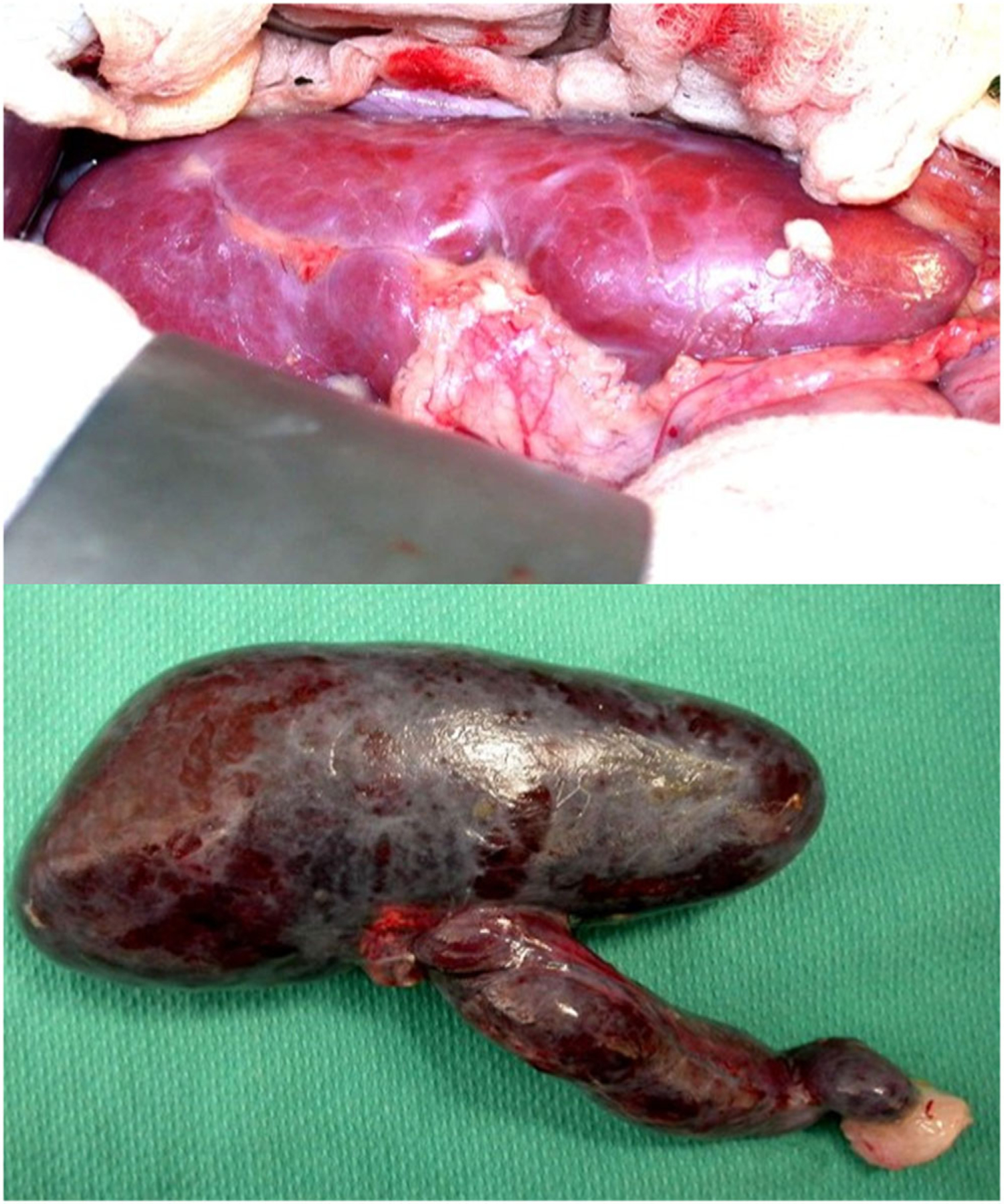
Top: A wild-type (genetically-unmodified) pig kidney immediately after its transplantation into a non-human primate recipient. It is a healthy pink color, indicating excellent blood flow. Bottom: The same kidney 5 min later. Immediate (hyperacute) rejection has taken place, consisting of thrombosis in the blood vessels, rupture of blood vessels, with hemorrhage into the tissues of the kidney, rendering it black

A major early problem in developing xenotransplantation was that, if an organ from a wild-type pig, that is, a genetically *unmodified* pig, was transplanted into a human or non-human primate, it stimulated an almost immediate immunological reaction that resulted in rejection, usually within minutes or hours (Figure 1) (Lexer et al., 1986; Ryczek et al., 2021). Therefore, it was necessary to genetically modify the pig to reduce the organ’s immunogenicity and increase its physiological compatibility (Cooper et al., 2016; Cowan & Tector, 2017). However, over the last 30 years researchers have made significant progress in overcoming many of these immunobiological barriers that had been hindering progress. One important milestone occurred in the early 1990s when a research team in Cambridge, UK, led by David White, generated transgenic pigs that expressed a human complement-regulatory protein (human decay-accelerating factor or CD55), which partially protected the pig organ from rejection after transplantation into non-human primates (Cozzi & White, 1995).

Following the development of gene-editing technology, it became possible to modify the pig genome by inserting or deleting genes (Ryczek et al., 2021). The ability to do this has been greatly facilitated by the introduction of the CRISPR/Cas9 technology (Jinek et al., 2012), However, it is not yet certain how many of these gene edits are essential in a single organ-source pig, though approximately 10 would appear to be particularly beneficial (Cooper et al., 2019).

However, using transgenic pig organs is not without its problems. For example, there are some significant physiological differences between pigs and humans (Hansen-Estruch et al., 2022). The physiological environment provided by the human body may not be optimal, which could affect the pig organ’s longevity. However, recent experiments of the transplantation of life-supporting genetically engineered pig kidneys into immunosuppressed non-human primates have extended survival for up to 499 days (Kim et al., 2019), suggesting that the function of the pig kidneys is satisfactory.

The prospect of a successful pig organ xenograft into a human is no longer merely theoretical. On January 7, 2022, a patient in the United States who was not a candidate for a human heart transplant received a pig heart, with the heart initially functioning well (Griffith et al., 2022). However, the patient died 2 months later probably from organ rejection (Cooper, 2022), though infection of the graft with porcine cytomegalovirus may have played a role (Mueller, 2022). Until that time, the use of pigs for clinical xenotransplantation had been deemed “safe” as pigs had previously been used in procedures involving the transplantation of islets of Langerhans, spleen, skin, cornea, and choroid plexus cells, with no serious complications or deaths (Hu et al., 2022). Nevertheless, the risk of a zoonotic infection is clearly possible (as illustrated previously). In addition, a small number of pig kidney transplant experiments have been carried out in brain-dead human subjects, but follow-up has been so short (~3 days) that no definitive conclusions can be drawn from them (Montgomery et al., 2022; Porrett et al., 2022).

It is not surprising that the United States is the leading proponent in developing pig kidney xenotransplantation. In 2019, 3 811 patients on the national kidney transplant waiting list died, and an additional 3 814 were removed from the list because they became too sick to receive a transplant (Nikzad et al., 2021). In addition, factors such as more permissive regulations involving novel therapies, and economic investment by the National Institutes of Health and US biotechnology companies, likely play a role. Pig kidneys, rather than hearts, have been suggested as the preferable organ for the first formal clinical trials because, if life-threatening complications arise, the pig kidney can be removed and dialysis can be recommenced (Jagdale et al., 2021; Cooper & Hara, 2021).

Formal clinical trials of pig kidney transplantation are likely to begin within the next few years, initially possibly as a bridge to a human kidney transplant, and eventually as destination therapy and this will be contingent on the results from the initial clinical trials.

## 5 | ELIGIBILITY FOR XENOTRANSPLANTATION CLINICAL TRIALS

The criteria for formal clinical trials for xenotransplantation remain a topic of continued debate (Hurst et al., 2022; Jagdale et al., 2021; Welin, 2000). Jagdale et al. (2021) and his colleagues have provided details of what they consider to be inclusion and exclusion criteria. Arguably, clinical trials should begin only after sufficient and consistent demonstration of life-supporting xenograft function in a preclinical model (Cooper et al., 2017). Despite recent advances, it is not clear that this threshold has been achieved, and a phase 1 clinical trial may not yet be justified.

### 5.1 | Potential risks to public health

For many years, the safety of xenotransplantation has been a concern in the scientific and bioethical community, especially the potential for zoonotic disease—infectious diseases that are transmitted from animals to humans—and the implications of this for public health (Boneva & Folks, 2004; Krishna & Lepping, 2011; Fishman, 2018). The potential risk must be carefully considered because microorganisms that are benign in one species can be fatal in others.

The major concern is not that a patient with a pig organ graft may become infected with a pig microorganism, but that the patient may spread the disease to other members of the community. Although unlikely, there is a potential for an epidemic and even pandemic. There is no shortage of examples of zoonoses that have caused major problems, for example, HIV, rabies, West Nile virus, Ebola, swine influenza, coronaviruses, Lassa fever, Marburg fever, and plague (Public Health England, 2019). Just 13 zoonotic diseases account for over 2 million human deaths and 2.4 billion cases of illness each year (Grace et al., 2012).

To our knowledge, until 2022, there had been no definite documented cases of zoonotic disease (Hu et al., 2022), or what is more accurately termed xenozoonosis, following the transplantation of pig tissues or cells into human recipients. However, the recent experience in the patient with a pig heart demonstrated that this is a possibility (Griffith et al., 2022). Importantly, pigs will be bred in biosecure pathogen-free environments that will greatly reduce, but may not completely prevent, the likelihood of the transfer of any potentially pathogenic microorganisms with the organ xenograft (Cooper et al., 2020).

One major concern has been the presence of porcine endogenous retroviruses (PERVs) in pigs. PERVs pose a unique risk because they are integrated into the genome of every nucleated cell in all pigs. Despite lacking known pathogenicity in pigs, it remains undetermined whether PERVs have the potential to become pathogenic in humans (Cengiz & Wareham, 2019). Under special laboratory conditions, PERVs have been able to infect certain human cells (Denner, 2018). However, transmission of PERVs has not been observed during preclinical xenotransplantation, but this may be associated with a difference in viral receptors in non-human primates. Scientists have deemed the risk posed to humans by PERVs to be low, but legitimate concerns remain about the risk of exposing the surrounding population to these viruses. Even a low risk to the public health is not necessarily an acceptable risk.

Althoughthe absolute risk of a xenozoonosis remains unknown, any health surveillance should be guided by the “precautionary principle.” The precautionary principle in public health describes the need to adopt precautionary measures when an intervention raises concerns about a risk to human health, in this case the risk of xenozoonosis during clinical trials of xenotransplantation. Therefore, despite the risk being low, considering the precautionary principle it would at the very least be necessary to ensure that appropriate public health surveillance measures are adopted during clinical trials. These might include, but are not limited to, the exhaustive screening of the source animal; routine screening of recipients pre- and post-xenotransplantation; and the routine evaluation of social and sexual contacts of xenotransplant recipients, and may possibly include the monitoring of household pets (Fishman et al., 2012). These requirements could be extremely demanding, and they raise concerns about whether a patient can give informed consent for this degree of medical oversight. Moreover, what happens if a participant in a clinical trial discontinues routine follow-up?

Fovargue and Ost (2010) have argued that—when considering the precautionary principle alongside John Stuart Mill’s harm principle (Mill, 1993)—xenotransplantation poses a risk so serious to the global population that it should be prohibited from moving to clinical trials. They define risk in this context as the “possible negative outcomes of xenotransplantation and the probability of these outcomes occurring,” and argue that the serious risk of an infectious pandemic outweighs any benefits that might accrue to individuals. This is because permitting clinical trials would knowingly expose the public health to some degree of risk that can neither be quantified, controlled, nor appropriately managed.

However, all potentially beneficial research has risks and it may be unreasonable to set a threshold so stringent that xenotransplantation is ruled out by default despite the substantial benefits it may bring to humanity. Importantly, even though the risks of a xenozoonosis have been minimized by inactivation the PERVs that are present in every pig cell (Niu et al., 2017), the risk of xenozoonosis will continue to remain unknown until clinical trials are permitted. The costs of not going ahead must also be factored into the application of the precautionary principle because, if xenotransplantation proves to be as effective as allotransplantation, then the long-term suffering that would be avoided would be substantial. Therefore, any decision to move toward clinical trials must balance the potential risks and costs against the potential benefits. The medical trajectory suggests that recruitment for formal clinical trials of solid organ xenotransplantation is likely to begin soon.

### 5.2 | Psychosocial considerations

The importance of psychosocial assessment and care of the recipients of human organs (allografts) is well established, though not always available to the patients (Maclean, 2018). It has been posited that a recipient of a pig xenograft could experience unique negative psychosocial effects that could affect their perception of self and how they are perceived by others. As an illustration, recipients of allografts have reported experiencing negative effects on their body image, for example, men with women’s hearts, though the prevalence and severity vary significantly, and this can lead to adverse effects that can compromise their recovery (Látos et al., 2012; Látos et al., 2015; Zimbrean, 2015). It is possible that receiving a xenotransplant may complicate and compound issues concerning body image and identity, resulting in psychosocial sequelae. Recipients of xenografts may therefore require additional psychological support both pre- and post-xenotransplantation.

Psychosocial concerns about xenotransplantation encompass a range of issues that include personality changes that could be brought about by receiving a xenograft, for example, (i) negative effects on relationships and how the recipient would be perceived by friends, family, and the public; (ii) the psychological impact of having an animal organ in their body and how it could affect their self-image; and (iii) the potential for shame and parental concerns about the bullying of their children should they receive a xenograft (De Bona et al., 2004; Hurst et al., 2021; Mohacsi et al., 1995; Padilla, Rhodes, et al., 2021; Stadlbauer et al., 2011).

The impact on children who are recipients of a xenograft could be profound. Despite attempts to maintain confidentiality, if it were disclosed that a child had a pig organ, parental concerns about bullying and its damaging psychological effects are likely well founded. However, despite the seriousness of these concerns, it is worth emphasizing that the alternative may be not surviving at all.

Importantly, the burden or concerns about the negative psychosocial effects will likely not be equally distributed. In the United States, African-American patients were more concerned about psychosocial issues and were less likely to accept a pig kidney xenograft compared with white patients (Padilla, Hurst, et al., 2021), though this may be associated with ethical abuse of African Americans in historic clinical studies. This raises further questions about exacerbating existing health inequalities and the potential challenges with ensuring fair access and representation in clinical trials.

### 5.3 | Animal welfare and rights

Ethical concerns about whether it is morally permissible to breed and raise genetically engineered pigs for the sole purpose of using them for their organs remains a topic of ongoing debate and disagreement (Bourret et al., 2016; Christoffersen, 2004; Drew, 2021; Manesh et al., 2014). In principle, pigs are already used in industrial farming for the purpose of food and clothing—indeed, more than 100 million pigs are slaughtered in the United States annually for food and 500 million in China to provide heparin. If this is permissible, then arguably raising them for their organs should be too. Is it less morally questionable to kill a pig to save a human life than to kill a pig for food, especially when alternative means of sustenance exist? This analogical argument is unlikely to be persuasive to supporters of animal rights given the moral gravity that many attach to industrial farming. Some philosophers have argued that if industrial farming is a serious moral evil because of the suffering it inflicts on animals, then killing them for less bad purposes does not necessarily entail it as morally permissible (Koplin, 2020).

Nevertheless, it remains generally accepted that it is morally permissible to induce some degree of harm to a non-human animal if sufficient benefits are accrued to humans, while ensuring that any harm to the animal is minimized or avoided, where possible. If this were not deemed to be morally permissible, then nearly all animal research required to be completed to assess drug discovery and safety before a formal clinical trial would not be acceptable. Until a comparable alternative was available, the impact on humanity would be substantial.

Pigs are social animals with emotional and cognitive capacities (Goumon & Špinka, 2016; Reimert et al., 2013) and so any intervention that requires killing them remains ethically contentious. It is possible that this could be exacerbated by the exponential growth of veganism today and vegan beliefs about the use of animals (The Vegan Society, 2022). This problem also raises further ethical questions about what kinds of environments the “donor” pigs will inhabit and the impact on their welfare and whether further suffering can be mitigated. In this respect, the pigs to be used as organ sources will be bred and housed under greatly superior conditions when compared with those maintained in industrial farming facilities.

## 6 | ECONOMIC CONSIDERATIONS

It is well documented that kidney transplantation is currently the optimal treatment for end-stage kidney disease. Not only are there notable health benefits for the patient, such as reduced morbidity and mortality, but there are also long-term societal economic savings. Spending on dialysis in the United States totals more than $30 billion per annum (United States Renal Data System, 2019). Treating chronic kidney disease in England is estimated to cost around £1.5 billion and accounts for over 1% of the National Health Service spending per annum (Kerr et al., 2012). If xenotransplantation is able to demonstrate clinical efficacy, it may provide significant cost savings, for example, by reducing the need for dialysis, and lead to the end of deceased donor kidney transplantation, which is expensive (Groth, 2007; Saari & Cooper, 2017; Bayliss, 2022). However, these perceived cost savings are predicated on pig kidney xenotransplantation being equally efficacious as allotransplantation, and currently that remains undetermined. Thus, any perceived economic cost savings on these grounds will be accurately determined only after the outcomes of formal clinical trials. Furthermore, any perceived cost savings may well be diminished when considering the need of for-profit biotechnology companies to recuperate the capital that will have been invested in the development of xenotransplantation, in some cases for more than 20 years. At this time, it therefore remains unclear whether xenotransplantation will result in a long-term economic cost saving.

### 6.1 | What do healthcare professionals think about xenotransplantation?

Because of the vital role that healthcare professionals have in caring for patients who either require or have been recipients of an organ transplant, researchers have identified the need to understand their views toward xenotransplantation. Recently, there have been several studies exploring the views of healthcare professionals toward xenotransplantation across a range of countries. As one would anticipate, there is some variation in opinion from country to country, but those surveyed tended to view the prospect of xenotransplantation favorably.

If the results of xenotransplantation are similar to those achieved after human organ transplantation (allotransplantation) and this, of course, is not yet known—74% (*n* = 6564) of student nurses (Martínez-Alarcón et al., 2019) and 76% (*n* = 112) of registered nurses (Conesa et al., 2006) in Spain would be in favor of initiating this form of therapy. In a smaller survey conducted in the United States (Padilla, Hurst, et al., 2021), student nurses demonstrated a similar attitude toward xenotransplantation but were concerned about the psychosocial effects on the recipient and the risk of infection that xenotransplantation might pose. Similarly, the majority of student nurses in Sweden, Turkey, and Poland viewed xenotransplantation favorably (Dogan et al., 2022; Hagelin et al., 2000; Mikla et al., 2016). Importantly, the further nursing students have progressed into their course, the more likely they are to view xenotransplantation favorably (Mitchell et al., 2020).

Similar patterns have been observed across a wider range of healthcare professionals involved in transplant-related care. For example, most transplant-related healthcare professionals surveyed in Spain, Cuba, and Mexico viewed xenotransplantation positively, although one third still viewed it negatively (Ríos et al., 2010). Furthermore, in a survey of nephrologists, transplant surgeons, and nurses involved in the care of renal transplant patients 80% had a positive view toward xenotransplantation, providing the risks and outcomes were similar to those of kidney allotransplantation (Padilla et al., 2020). The general pattern is that those healthcare professionals involved in the direct care of renal patients tend to have a slightly more positive view of xenotransplantation compared to those who do not.

## 7 | CONCLUSION

In summary, a shortage of kidneys to meet the global demand has driven research into alternative means of sourcing organs for transplantation. Recent developments in gene-editing technology have helped to reinvigorate xenotransplantation research and it is now the most likely alternative to move into formal clinical trials in the not-too-distant future. Arguably the unanswered questions regarding its efficacy can now only really be answered by clinical trials. Nevertheless, despite these recent advances there remain concerns and unanswered questions about the potential public health risk it poses, as well as other ethical issues that will rightly continue to be debated. Despite the mostly favorable views of healthcare professionals toward xenotransplantation, higher education institutions may play an important role in ensuring that they are suitably informed about its continued development both for their own education and for that of their patients.

## Figures and Tables

**TABLE 1:** World experience in kidney transplantation from animals to humans (clinical kidney xenotransplantation)

Year	Surgeon	Source animal	Number of transplants	Patient maximal survival (days)	Reference
1905	Princeteau	Rabbit (kidney slices)	1	16	Princeteau (1905)
1906	Jaboulay	Pig	1	3	Jaboulay (1906)
		Goat	1	3	
1910	Unger	Monkey	1	2	Unger (1910)
1913	Schonstadt	Monkey	1	Not stated	Morel and Papin (1913)
1923	Neuhof	Sheep	1	9	Neuhof (1923)
1964	Reemtsma	Chimpanzee	6	<9 months	Reemtsma et al. (1964)
		Monkey	1	10	
1964	Hitchcock	Baboon	1	5	Hitchcock et al. (1964)
1964	Starzl	Baboon	6	<60	Starzl et al. (1964)
1964	Hume	Chimpanzee	1	1	Hume (1964)
1964	Traeger	Chimpanzee	3	<49	Traeger et al. (1965)
1965	Goldsmith	Chimpanzee	2	<4 months	JAMA (1985)
1966	Cortesini	Chimpanzee	1	31	Cortesini et al. (1970)
1966	Kuss	Pig	1	2	Kuss (1991)

**TABLE 2:** The advantages of the pig as a potential source of organs and cells for humans\

Availability	Unlimited
Breeding potential	Good
Period to reproductive maturity	4–8 months
Length of pregnancy	114 ± 2 days
Number of offspring	5–12
Growth	Rapid (adult human size within 6 months)^a^
Size of organs for all ages of humans	Adequate
Anatomical similarity to humans	Close
Physiological similarity to humans	Close
Relationship of immune system to humans	Distant
Knowledge of tissue typing	Considerable (in selected herds)
Blood type compatibility with humans	ABO-blood type compatibility can be assured (All pigs will be of type O [non-A])
Experience with genetic engineering	Considerable
Risk of transfer of infection (xenozoonosis)	Low
Availability of specific pathogen-free animals	Yes
Cost of maintenance	Under the biosecure designated pathogen-free conditions required by the national regulatory authorities the costs will be significant.
Public opinion	Generally supportive

a Various miniature pigs reach a maximum weight of 10%–50% of the weight of domestic pigs.

**TABLE 3:** Potential advantages of pig kidney xenotransplantation over allotransplantation (if the immunological challenges can be successfully overcome)

1. Unlimited supply of “donor” organs
2. Organs available electively, that is, whenever required. (Patients with end-stage organ failure will be able to receive a transplant immediately, without any need for such supportive therapies as dialysis, mechanical circulatory support, or intensive care)
3. Avoids the detrimental effects of brain death on the donor organs (which can cause structural injury to an organ and/or early metabolic dysfunction after transplantation)
4. The “donors” will be free of all potentially infectious microorganisms (and of endogenous retroviruses, if necessary)
5. “Borderline” transplant candidates, that is, those with health problems that may be detrimental to prolonged patient survival after organ transplantation, for example, poorly controlled diabetes, severe peripheral or cerebral vascular disease, will be more acceptable (as they will no longer be competing for scarce organs with other potential transplant candidates)
6. Avoids the cultural barriers to deceased human organ donation that are present in some countries, for example, Japan.

## Key points

- There is a global shortage of organs and xenotransplantation may one day contribute to alleviating it.
- Recent scientific breakthroughs mean that formal clinical trials are likely to begin soon, moving us one step closer to a future where people may no longer die while waiting for a kidney transplant.
- Xenotransplantation remains contentious because of ethical concerns around the use of non-human animals and its potential public health risks.
